# Exosomes from high glucose-treated glomerular endothelial cells activate mesangial cells to promote renal fibrosis

**DOI:** 10.1242/bio.015990

**Published:** 2016-03-23

**Authors:** Xiao-ming Wu, Yan-bin Gao, Fang-qiang Cui, Na Zhang

**Affiliations:** Beijing Key Lab of TCM Collateral Disease Theory Research, School of Traditional Chinese Medicine, Capital Medical University, No.10, Youanmenwai, Xitoutiao, Fengtai District, Beijing 100069, China

**Keywords:** Exosome, Glomerular endothelial cells, Glomerular mesangial cells, High glucose, Activation, Renal Fibrosis

## Abstract

The interaction between glomerular endothelial cells (GECs) and glomerular mesangial cells (GMCs) is an essential aspect of diabetic nephropathy (DN). Therefore, understanding how GECs communicate with GMCs in the diabetic environment is crucial for the development of new targets for the prevention and treatment of DN. Exosomes, nanometer-sized extracellular membrane vesicles secreted by various cell types, play important roles in cell-to-cell communication via the transfer of mRNA, microRNA and protein. In this study, we demonstrate that high glucose (HG)-treated GECs secrete a higher number of exosomes highly enriched in TGF-β1 mRNA compared with normal glucose (NG)-treated GECs. Exosomes released by HG-treated GECs can promote α-smooth muscle actin (α-SMA) expression, proliferation and extracellular matrix protein overproduction in GMCs through the TGF-β1/Smad3 signaling pathway. Thus, we provide new insights into the pathogenesis of DN that involves intercellular transfer of TGF-β1 mRNA in the GEC-to-GMC direction via exosomes.

## Abstract (precis)

**Summary:** In this study, we demonstrate that TGF-β1-containing exosomes from high glucose-treated glomerular endothelial cells can activate glomerular mesangial cells to promote renal fibrosis.

## INTRODUCTION

Diabetic nephropathy (DN), one of diabetic microvascular complications, is the leading cause of end-stage renal failure requiring dialysis and/or kidney transplantation (Ritz et al., 1999). Glomerulosclerosis is a predominant pathologic feature of DN and characterized by accumulation of extracellular matrix (ECM), glomerular basement membrane thickening, and glomerular capillaries obliteration (Dronavalli et al., 2008; Simonson, 2007). Overproduction of matrix proteins is considered to be due to the phenotypic modulation of the glomerular mesangial cells (GMCs) (Johnson et al., 1992). GMCs, specialized microvascular pericytes, form the central stalk of the glomerulus that provide structural support for glomerular capillary loops (Armulik et al., 2005). In diabetes, in response to metabolic, immunologic or hemodynamic injury, GMCs acquire an activated phenotype, characterized by an induction of α-smooth muscle actin (α-SMA), and undergo hypertrophy, proliferation with overproduction of ECM (Abboud, 2012). Among those molecular mediators of GMCs activation, transforming growth factor- β1 (TGF-β1) is a central profibrotic cytokine that elevated significantly in diabetes and strongly induces α-SMA and ECM production in GMCs (Dai and Liu, 2004). TGF-β1 exerts profibrotic effects in GMCs by initiating canonical and non-canonical pathways. Among them, TGF-β1/Smad3 signaling is demonstrated to be a major pathway in activation of GMCs (Meng et al., 2015).

In the glomerulus, GMCs and GECs are in direct contact and interact closely with each other. Alterations in GECs can produce changes in GMCs under both physiological and pathophysiological conditions (Schlondorff and Banas, 2009). For example, GECs-derived platelet-derived growth factor B (PDGFB) can recruit mesangial cells into the developing glomeruli and promote capillary loops formation (Bjarnegard et al., 2004). In diabetes, GECs dysfunction, one of the earliest events, is already present in the normoalbuminuric stage of diabetes and likely contribute to DN by releasing paracrine mediators to cause GMCs damage (Dimke et al., 2015; Fu et al., 2014). Identification of the mechanism of intercellular communication between GECs and GMCs in diabetes will lead to a better understanding of the pathogenesis of DN, however, which remains to be elucidated.

Recently, exosomes, nanometer-sized extracellular membrane vesicles secreted by various cell types, have been considered to play important roles in cell-to-cell communication via the transfer of their cargo, such as proteins, mRNA or microRNA (Corrado et al., 2013). Existing research shows that endothelial cells can secret exosomes which are able to enter target cells and alter cell function. For example, cardiac endothelial cell-derived exosomes can induce B cells release TGF-β and suppress effector T cell proliferation (Song et al., 2014). Endothelial exosomes loaded with miR-503 can inhibit tumor cell proliferation and invasion during breast cancer neoadjuvant chemotherapy (Bovy et al., 2015). Recent studies indicate that in pathologic conditions, some cells-derived exosomes have the capacity to trigger phenotypic change of target cells by transferring TGF-β protein or mRNA. For example, some cancer-derived exosomes rich in TGF-β can trigger differentiation of fibroblast towards a myofibroblastic phenotype (Webber et al., 2010). Hypoxic epithelial cells secrete a significantly higher number of TGF-β1 mRNA-containing exosomes that has the capacity to initiate activation and proliferation of fibroblasts (Borges et al., 2013). However, in diabetic environment whether GECs-derived exosomes could enter nearing GMCs and activate GMCs is completely unknown.

Diabetes mellitus (DM) is characterized by increased blood glucose levels. In this study, we examined whether exposure of GECs to high glucose (HG) could induce release of exosomes and we examined whether exosomes from HG-treated GECs trigger activation of GMCs to promote renal fibrosis *in vitro* and *in vivo*.

## RESULTS

### HG-treated GECs secrete a higher number of exosomes compared with NG-treated GECs

**Fig. 1.**
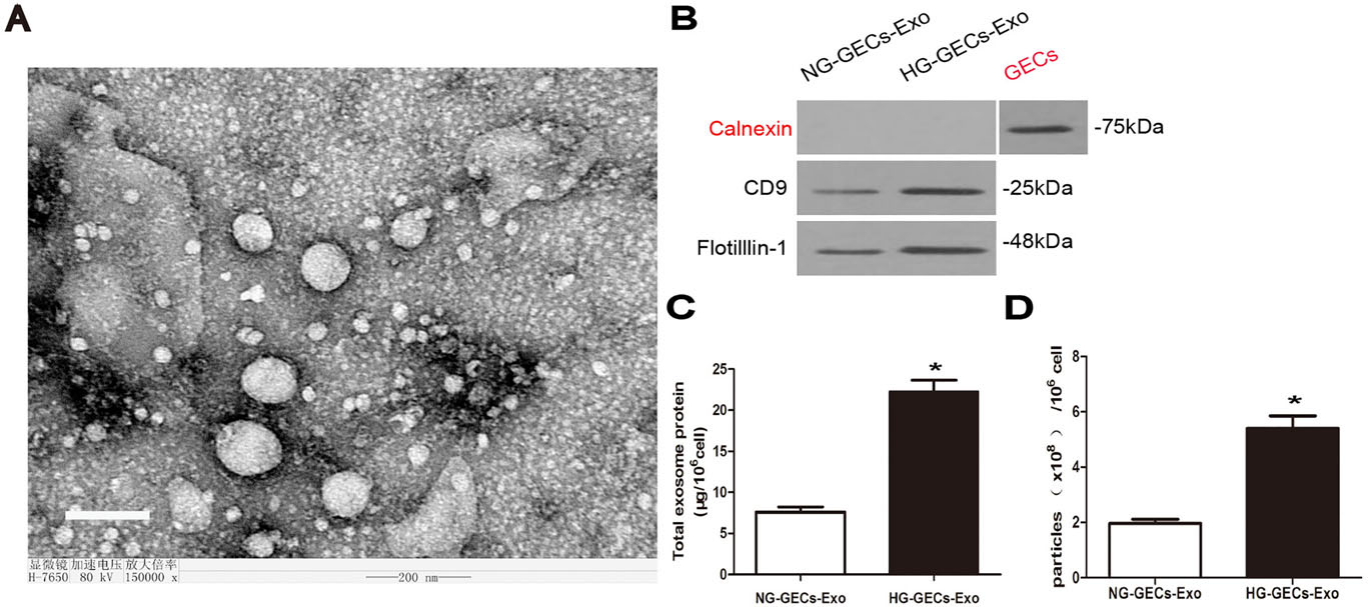
**An increased number of exosomes are released by HG-treated GECs.** (A) Exosomes extracted from GECs were identified by TEM. Magnification: ×150,000. Scale bar: 200 nm. (B) Protein blot of exosomes derived from 4×10^6^ NG-treated or HG-treated GECs using CD9 antibody, Flotillin-1 antibody and calnexin antibody. (C) Quantification of total exosome protein. (D) Quantification of exosomes extracted from NG-treated or HG-treated GECs. NG-GECs-Exo, exosomes from NG-treated GECs. HG-GECs-Exo, exosomes from HG-treated GECs. Mean±s.d.; *n*=3; *P<0.05, significantly different from NG-treated GECs derived exosomes.

To examine the impact of HG on exosomes release, GECs were cultured under normal glucose (NG; 5.5 mmol/l glucose+24.5 mmol/l mannitol) and HG conditions (30 mmol/l) for 24 h. Exosomes were isolated from 5 ml culture supernatants of approximately 4×10^6^ GECs as described in Materials and methods. First we examined samples by transmission electron microscopy (TEM), which revealed the presence of vesicles ranged in size from 30 nm to 100 nm (Fig. 1A). To examine the purity of isolated exosomes, we performed western blotting to detect the presence of exosomal marker CD9 and lipid-raft marker Flotillin-1 as well as the absence of calnexin which is a marker of endoplasmic reticulum and often associated with cell debris (Lee et al., 2013; Emanueli et al., 2015). As expected, calnexin was not detected in isolated exosomes, demonstrating that the lack of cellular components and debris in our exosomes preparations. Furthermore, CD9 and Flotillin-1 were detected in exosomes and their expression was higher in exosomes preparations from HG-treated GECs compared with NG-treated GECs (Fig. 1B).

To determine whether HG-treated GECs release more exosomes, relative total protein content was measured. Total exosome protein normalized by cell number was significantly higher when isolated from HG-treated GECs (Fig. 1C). To evaluate the number of exosomes released by GECs in NG and HG conditions, we quantified exosomes as described in Materials and methods. The results indicated that HG-treated GECs secreted a higher number of exosomes compared with NG-treated GECs (Fig. 1D).

Taken together, these findings confirm the presence and purity of GECs-derived exosomes in our preparations and indicate that HG promotes the release of exosomes by GECs.

### GECs-derived exosomes are internalized by GMCs

**Fig. 2.**
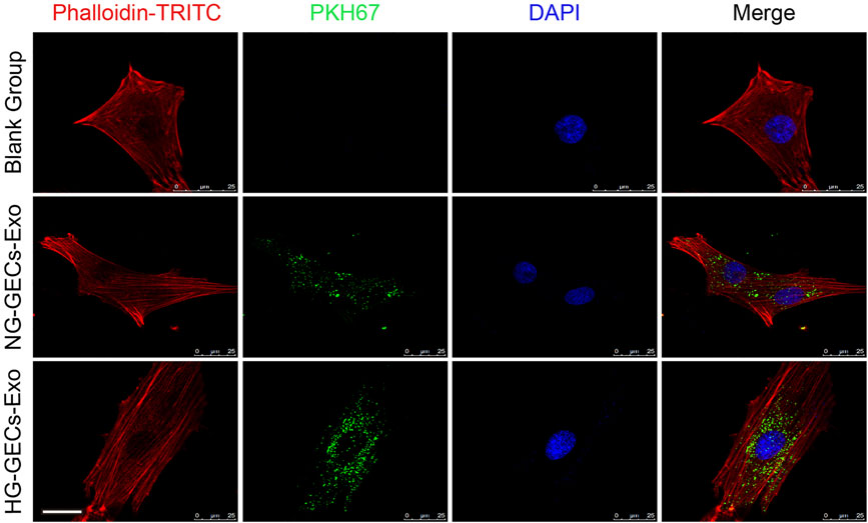
**PKH67-labeled glomerular endothelial exosomes can be internalized by GMCs.** Exosomes isolated from culture supernatants of NG treated- or HG treated-GECs were labeled with fluorescent dye PHK67 and incubated with GMCs for 24 h. Then the GMCs were stained with phalloidin for intracellular cytoskeleton f-actin. Scale bars: 25 μm.

To study the internalization of GECs-derived exosomes by GMCs, we labeled GECs derived exosomes with a green fluorescent marker, PKH67, and incubated GMCs with the labeled exosomes for 24 h. Then the GMCs were stained with phalloidin for intracellular cytoskeleton f-actin. Cellular uptake of GECs-derived exosomes by GMCs was observed under the confocal laser microscopy (Fig. 2). We found that PKH67-labeled exosomes were localized in the cytoplasm of GMCs, implying that GECs-derived exosomes can be internalized by GMCs.

### HG-treated GECs derived exosomes induce GMCs activation, proliferation and matrix overproduction *in vitro* and *in vivo*

**Fig. 3.**
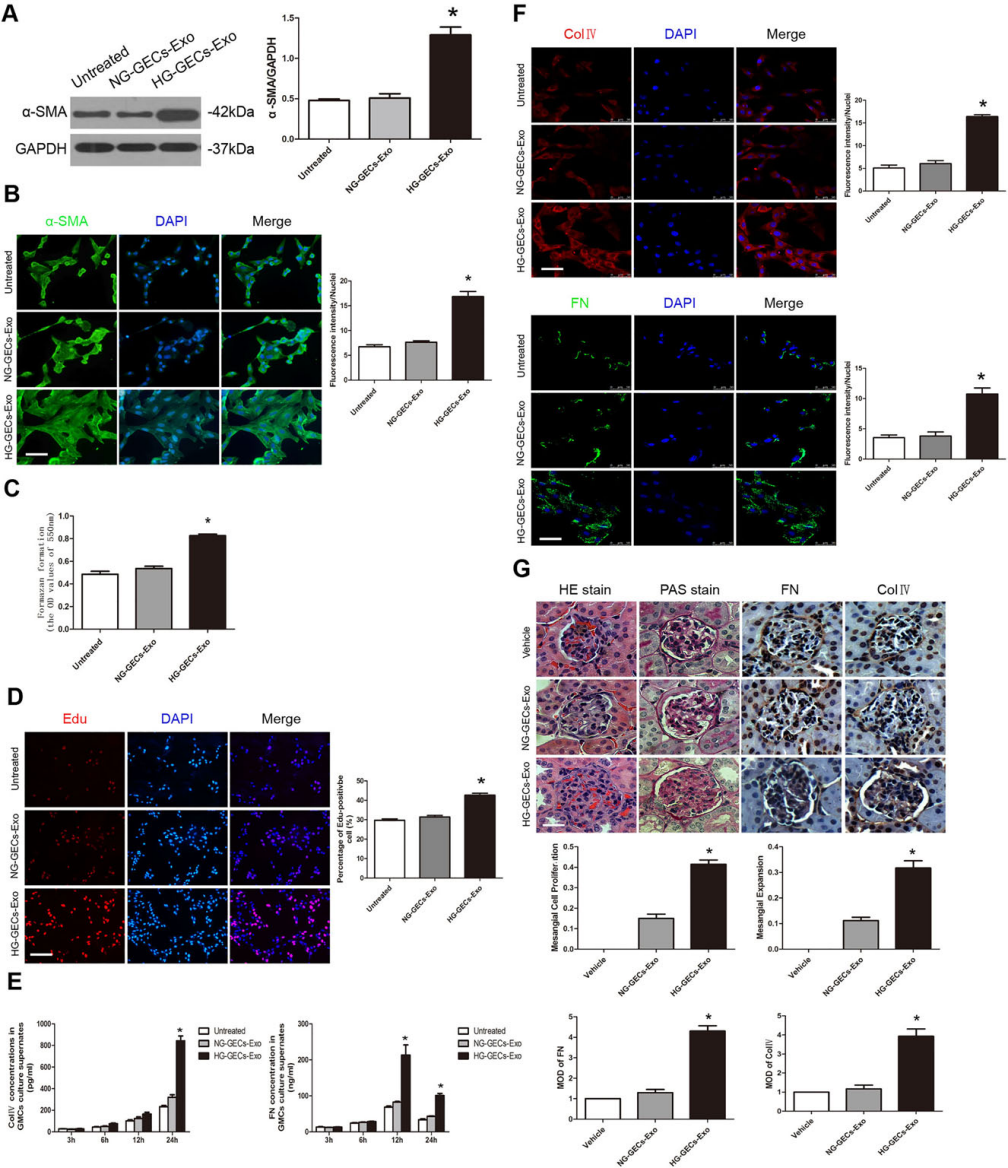
**HG-induced glomerular endothelial exosomes lead to GMCs activation and subsequent GMCs proliferation and ECM overproduction.** (A,B) α-SMA expression in GMCs co-cultured with exosomes released by NG- or HG-treated GECs was assessed by western blot and immunofluorescence staining. Scale bars: 50 μm. (C,D) MTT assay and Edu staining showed proliferation of GMCs exposed to exosomes derived from NG- or HG-treated GECs. Scale bars: 100 μm. (E,F) Extracelluar and intracelluar ColIV and FN was evaluated after GMCs co-incubation with exosomes for 24 h by ELISA and immunofluorescence staining respectively. Scale bars: 50 μm. (G) The percentage of glomeruli showing mesangial proliferation and expansion was assessed in the mice injected with exosomes from NG- or HG-treated GECs. The mean optical density (MOD) of FN and ColIV proteins was evaluated in the mice. Scale bars: 50 μm. Mean±s.d.; *n*=3-6; *P<0.05, significantly different from untreated GMCs.

α-Smooth muscle actin (α-SMA) is the marker which can differentiate between ‘activated’ and resting GMCs (Dai and Liu, 2004). To investigate whether the exosomes secreted by HG-treated GECs activate GMCs, exosomes derived from 1×10^6^ NG- or HG-treated GECs were added to cultures of GMCs. FBS-deprived GMCs were treated for 24 h with exosomes. Exosomes from HG-treated GECs but not NG-treated GECs specifically triggered α-SMA expression in cultured GMCs as assessed by western blot analysis (Fig. 3A). Similar results were obtained by using an immunofluorescence staining. Treatment of GMCs with exosomes derived from HG-treated GECs for 24 h significantly induced α-SMA expression (Fig. 3B).

To determine whether exosomes from HG-treated GECs induce proliferation of GMCs, we performed MTT assay and 5-ethynyl-2′-deoxyuridine (EdU) staining respectively. A significant increase of cell proliferation was observed in GMCs co-cultured with exosomes released by HG-treated GECs (Fig. 3C,D).

One of the consequences of GMCs activation is to overproduce ECM (Dai and Liu, 2004). To examine the effects of exosomes on induction of ECM components in GMCs, we performed enzyme-linked immunosorbent assay (ELISA) for the quantitative determination of type IV collagen (ColIV) and fibronectin (FN) concentrations in GMCs culture supernates at different time points. The results revealed that co-incubation of GMCs with exosomes from HG-treated GECs but not NG-treated GECs, increased ColIV and FN expression compared with untreated GMCs in a time-dependent manner (Fig. 3E). Consistently, immunofluorescence staining showed that exosomes released by HG-treated GECs but not NG-treated GECs induced intracellular ColIV and FN accumulation in cultured GMCs (Fig. 3F).

To evaluate whether glomerular endothelial exosomes derived under HG conditions cause GMCs proliferation and mesangial expansion *in vivo*, we generated endothelial exosomes *in vitro* from 1×10^5^ HG-treated GECs. Control exosomes were purified from the supernatant of 1×10^5^ NG-treated GECs. C57BL/6 mice were injected via the tail vein with exosomes derived from NG- or HG-treated GECs or vehicle 5 times per week for 3 weeks. Then the kidneys were harvested and pathological changes were observed by hematoxylin and eosin (H&E), periodic acid–Schiff (PAS) staining and immunohistochemistry. Compared with mice in the control groups, HG-induced glomerular endothelial exosomes treated mice displayed a significantly mesangial expansion, proliferation and ECM protein overproduction. (Fig. 3G).

### Activation of GMCs by HG-induced glomerular endothelial exosomes is TGF-β1/Smads signaling pathway-dependent

**Fig. 4.**
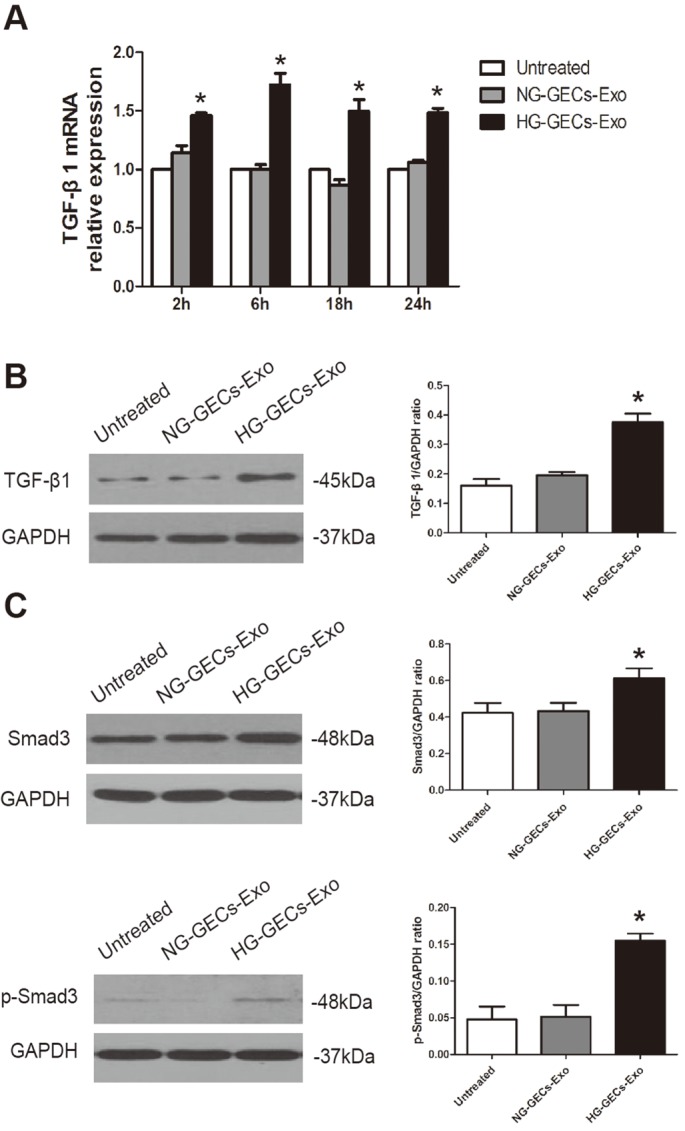
**HG-induced glomerular endothelial exosomes activate TGF-β1/Smads signaling pathway in GMCs.** (A,B) GMCs were co-incubated with HG-induced glomerular endothelial exosomes for 2, 6, 18, and 24 h, and then TGF-β1 expression in GMCs was analyzed by real time RT-PCR and western blot, respectively. (C) p-Smad3, the phosphorylated and active form of Smad3 and Smad3 expression was assessed by western blot. Mean±s.d.; *n*=4; *P<0.05, significantly different from untreated GMCs.

TGF-β1 is the most potent cytokine that induces mesangial cells activation both *in vitro* and *in vivo* (Chen et al., 2001; Schnaper et al., 2003; Bottinger and Bitzer, 2002; Border and Noble, 1997). To assess whether TGF-β1 is involved in exosomes-induced activation of GMCs and upregulation of ECM proteins, we performed real-time RT-PCR analysis on TGF-β1 in GMCs after exosomes stimulation at different time points. HG-induced endothelial exosomes treated GMCs showed a higher expression of TGF-β1 mRNA compared with control endothelial exosomes treated and untreated GMCs (Fig. 4A). Western blot analysis confirmed that co-incubation of GMCs with HG-induced endothelial exosomes for 24 h, but not control endothelial exosomes, increased TGF-β1 expression compared with untreated GMCs (Fig. 4B).

Next, we aimed to explore a possible pathway by which HG-treated GECs derived exosomes trigger activation of GMCs. Smad signaling is demonstrated as a major pathway of TGF-β signaling in DN and Smad3 is highly activated during fibrogenesis (Meng et al., 2015). To examine whether Smad signaling is involved in exosomes-induced activation of GMCs, we performed western blot analysis on Smad3 and its active form phospho-Smad3 (p-Smad3) after exosomes stimulation. The data revealed that treatment of GMCs with exosomes released from HG-treated GECs but not NG-treated GECs increased Smad3 expression and activated Smad3 to p-Smad3 (Fig. 4C).

### HG-induced glomerular endothelial exosomes derived TGF-β1 mRNA is functionally important for the activation of GMCs

**Fig. 5.**
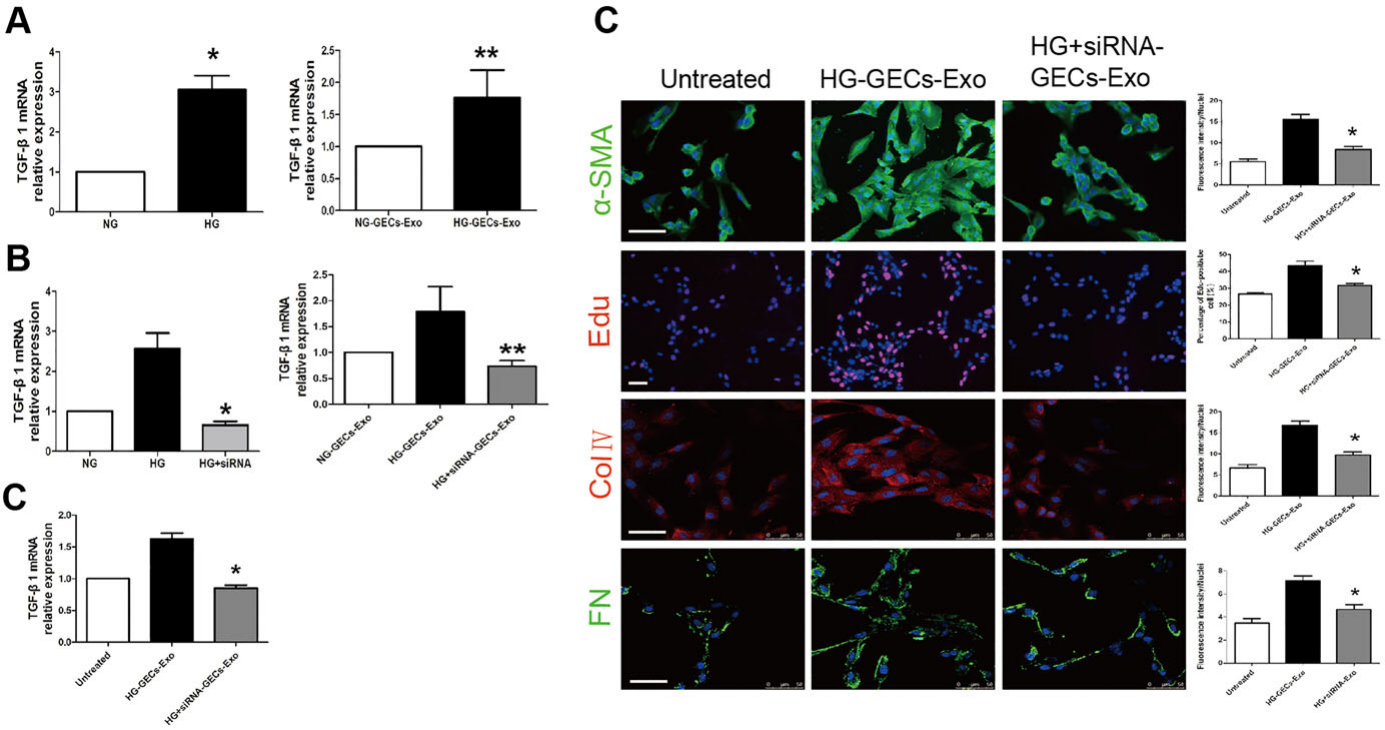
**TGF-β1 expression is increased in HG-treated GECs and exosomes derived from them.** Exosomes released from HG-treated GECs with TGF-β1 siRNA fail to activate GMCs. (A) TGF-β1 expression in GECs and GECs derived exosomes under NG or HG conditions. *P<0.05 compared with GECs under NG condition (*n*=4). **P<0.05 compared with NG-treated GECs derived exosomes (*n*=5). (B) TGF-β1 expression in GECs and GECs derived exosomes under NG, HG or HG+siRNA conditions. In HG+siRNA group, GECs are treated with TGF-β1 siRNA, exposed to high glucose for 24 h, and exosomes are obtained for TGF-β1 expression analyses. *P<0.05 compared with HG-treated GECs; **P<0.05 compared with HG-treated GECs derived exosomes. (C) When GMCs are co-cultured with control endothelial exosomes, HG induced endothelial exosomes and exosomes silenced for TGF-β1 mRNA, TGF-β1 expression, α-SMA expression, ColIV expression, FN expression and cells proliferation of GMCs are assessed by real time RT-PCR and immunofluorescence. HG+siRNA-GECs-Exo, exosomes extracted from GECs treated with TGF-β1 siRNA for 24 h of HG. *P<0.05 compared with GMCs incubated with HG-treated GECs derived exosomes (*n*=3-4). Scale bars: 50 μm.

We sought to investigate which factor in exosomes might be responsible for activation of GMCs. In diabetic conditions, hyperglycaemia-induced overproduction of TGF-β1 is causally associated with the development of DN (Meng et al., 2015). First, we performed real-time RT-PCR analysis to examine whether HG could increase the expression of TGF-β1 mRNA in GECs and GECs derived exosomes. After incubation with 30 mmol/l HG for 24 h, GECs and GECs-derived exosomes showed significantly increased expression of TGF-β1 mRNA compared with NG-treated GECs and exosomes derived from them (Fig. 5A). The data showed that the exosomes derived from HG-treated GECs might activate GMCs by transferring TGF-β1 mRNA.

Next, we used TGF-β1 small interfering RNA (siRNA) to silence TGF-β1 in GECs and a significant decrease in TGF-β1 mRNA expression was observed in HG-treated GECs with TGF-β1 siRNA and the GECs derived exosomes (Fig. 5B). When GMCs were treated with exosomes silenced for TGF-β1 for 24 h, TGF-β1 expression, α-SMA expression, the proliferation of cells, ColIV expression and FN expression remained unchanged, indicating that GMCs activation did not occur (Fig. 5C). The results suggested that TGF-β1 mRNA derived from HG-induced glomerular endothelial exosomes probably mediate GMCs activation.

## DISCUSSION

In diabetes, abnormal interaction of GECs and GMCs has been considered as an essential aspect of the pathogenesis of DN. However, until now, the research for communication between GECs and GMCs has been scarce. Recently exosomes have emerged as a novel and important mechanism of intercellular communication. Notably, extensive research has demonstrated that in many pathological conditions, injured cell-derived exosomes can alter phenotype of target cells by transferring mRNA, microRNA and protein-based transcription factors. But, whether exosomes from HG-treated GECs could enter adjacent GMCs and lead to the phenotypic change of GMCs has not been explored. In this study, we demonstrate that HG-treated GECs are able to produce a significantly higher number of exosomes compared with NG-treated GECs. Glomerular endothelial exosomes can be taken up into GMCs. Exosomes extracted from HG-treated GECs, but not from NG-treated GECs, trigger activation of GMCs and subsequent cells proliferation and overproduction of ECM both *in vitro* and *in vivo*. TGF-β1/Smad3 signaling is actively involved in the exosomes-induced activation of GMCs. Furthermore, our findings suggest that exosomes derived from HG-treated GECs may cause GMCs activation by the delivery of TGF-β1 mRNA. Our identification of this TGF-β1-containing exosome-mediated GECs-GMCs crosstalk may provide a basis for more effective therapeutic strategies to prevent DN.

In multicellular organs, cells continuously secrete exosomes containing mRNAs, miRNAs, proteins and lipids to communicate with each other under normal conditions (Robbins and Morelli, 2014). However, some harmful stimulating factors, such as pressure overload (Pironti et al., 2015), oxidative stress (Hedlund et al., 2011), hypoxia (Borges et al., 2013), acidic PH (Parolini et al., 2009), heat shock (Clayton et al., 2005), alcohol (Momen-Heravi et al., 2015) and so on, can alter the quantity and content of cell-derived exosomes which lead to abnormal interaction with target cells. Diabetes mellitus (DM) is characterized by hyperglycemia which has cytotoxic effect on endothelial cells (Liu et al., 2015). In this study, we show that HG condition not only induce GECs to release a higher number of exosomes, but also increase the TGF-β1 mRNA level in the exosomes. We also demonstrate that the changing trend of TGF-β1 mRNA in the exosomes is consistent with that of the producing cells. Nevertheless, we cannot exclude other HG-driven proteins, mRNAs or microRNAs in the exosomes that could potentially play a role in intercellular communication.

A lot of research has proven that exosomes can be incorporated by target cells (Skog et al., 2008). In our study, we first show that membrane dye PKH67 labelled exosomes released by GECs can be internalized by GMCs. Therefore, TGF-β1 mRNA is delivered from HG-treated GECs to the neighboring GMCs by exosomes, and GMCs in turn translate it to TGF-β1 protein, leading to elevated expression of TGF-β1 mRNA and protein in GMCs as shown in our study. TGF-β1, a central mediator in renal fibrosis, through Smad3, but not Smad2, to exert its fibrotic activities on GMCs (Meng et al., 2015). In our study, we demonstrate that TGF-β1 containing exosomes are able to change phenotype of GMCs, induce cells proliferation and overproduction of ECM, which are present in DN (Makino et al., 1995). But, when HG-treated GECs are incubated with TGF-β1 siRNA, a significant decrease in TGF-β1 mRNA expression was observed in the GECs and the extracted exosomes. Exosomes silenced for TGF-β1 cannot activate GMCs, supporting the fact that transferred TGF-β1 mRNA by exosomes is functionally important in the intercellular communication between GECs and GMCs.

In conclusion, this is the first report of a mRNA transferred from GECs to GMCs by the means of exosomes. We demonstrate that TGF-β1-containing exosomes from HG-treated GECs can activate GMCs to promote renal fibrosis. Our findings provide new insights into the pathological mechanism of DN.

## MATERIALS AND METHODS

### Cell culture

Mouse primary kidney glomerular endothelial cells (C57-6014G) from Cell Biologics were cultured in endothelial cell medium supplemented with 5% FBS. Mouse glomerular mesangial cells (SV40 MES 13) from China Infrastruture of Cell Line Resources were cultured in low glucose Dulbecco's Modified Eagle's Medium (DMEM) supplemented with 5% FBS. The cells were incubated at 37°C, with 5%CO_2_.

### *In vivo* studies

To specifically examine the role of HG-induced endothelial exosomes, eight-week-old male C57BL/6 mice (Academy of Military Medical Sciences, Beijing, China) were injected via the tail vein with exosomes generated *in vitro* by 1×10^5^ NG- or HG-treated GECs or an equal volume of PBS as a vehicle control five times per week for 3 weeks. The study protocol was approved by the Institutional Animal Care and Use Committee at Capital Medical University.

### Cell treatments and transfections

For the normal glucose treatment, mannitol was added to the culture medium (24.5 mM final concentration). For the high glucose treatment, D-Glucose was added to the culture medium (30 mM final concentration). For siRNA experiment, GECs were transfected with TGF-β1 siRNA (GenePharma Technology) using lipofectamine (Invitrogen) according to the manufacturer instructions.

### Exosome extraction

GECs were cultured under different conditions at 37°C for 24 h. Exosomes were isolated using exosome precipitation reagent ExoQuick-TC (System Biosciences) as previously described (Alvarez et al., 2012). Briefly, cell culture supernatants were collected and centrifuged at 3000 ***g*** for 15 min to remove cells and cell debris. 3.3 ml of ExoQuick-TC Exosome Precipitation Solution was added to 10 ml of the supernatants and the mixture was refrigerated overnight. Then, the mixture was centrifuged at 10,000 ***g*** for 30 min and the supernatants were aspirated. The residual solution was centrifuged at 1500 ***g*** for 5 min and removed. The purified exosomes were resuspended in PBS for direct use in subsequent experiments.

### Western blot analysis

Protein samples of cells and exosomes were extracted, subjected to SDS-polyacrylamide gel electrophoresis and then transferred to PVDF membranes. The PVDF membranes were blocked with 5% skim milk in PBS+0.05% Tween-20 and treated with the indicated antibodies at 4°C overnight, washed and then incubated with the secondary antibodies conjugated to horseradish peroxidase (HRP) for 2 h. Bands were detected with enhanced chemiluminescence (ECL) and then exposed to X-ray. Densitometry was detected by ImageJ (NIH). Immunoblotting was performed with mouse monoclonal to CD9 antibody (1:500; Abcam), rabbit monoclonal to Flotillin 1 antibody (1:10,000; Abcam), mouse monoclonal to Calnexin antibody (1:200; Abcam), rabbit polyclonal to α-SMA antibody (1:1000; Abcam), mouse monoclonal to TGF-β1 antibody (1:1000; Abcam), rabbit monoclonal to Smad3 antibody (1:1000; Abcam), rabbit monoclonal to p-Smad3 antibody (1:2000; Abcam).

### TEM

Exosomes were analyzed by transmission electron microscopy using negative staining. 2 μl of exosomes was placed on Formvar/carbon coated copper mesh grids, washed three times with PBS, and fixed with 2.0% phosphotungstic acid in aqueous suspension. Samples were examined with a Hitachi 7100 transmission electron microscope.

### Quantification of exosome particles

We quantified and compared the number of exosome particles using the EXOCET Exosome Quantitation Kit and CD63 ELISA kit (System Biosciences) according to the manufacturer's instructions as previously described (Alvarez et al., 2012).

### PKH67 labeling of exosomes and phalloidin staining of cells

Exosomes were labeled with PKH67 dye (Sigma) according to the manufacturer's instructions, and incubated with cells for 24 h. Then the cells were stained with phalloidin (Sigma) for intracellular cytoskeleton f-actin and observed under the confocal laser microscope.

### MTT assay and Edu labeling

GMCs were seeded in 96-well plates at a concentration of 5×10^4^ cells/well. The cells were incubated in a cell culture incubator overnight and then treated with exosomes for 24 h. MTT solution (5 mg/ml) was added at a volume of 10 μl in each well and was incubated for 4 h. Absorbance values were measured at the wavelength of 570 nm.

Proliferation of GMCs was also measured by Click-iT Edu Alexa Fluor 594 Imaging Kit (red fluorescence, Life Technologies) according to the manufacturer's instructions (Yu et al., 2009).

### Immunofluorescence

Cells were grown on 24-well chamber slides and incubated with exosomes for up to 24 h. Cells were washed three times with PBS, fixed with 4% paraformaldehyde, permeabilized with 0.1% Triton X-100, blocked with 5% normal goat serum to minimize non-specific binding. Then cells were sequentially incubated with primary antibodies overnight at 4°C and with secondary antibodies for 1 h at 37°C. Nuclei were stained with 4′,6-diamidino-2-phenylindole (DAPI). Coverslips were mounted on glass slides with glycerin and viewed using Nikon fluorescence microscopy and confocal microscope. Images were captured using a CCD camera and analyzed using Metamorph software (Molecular Devices). Immunofluorescence was performed with rabbit polyclonal to α-SMA antibody (1:50; Abcam), rabbit polyclonal to Fibronectin antibody (1:200; Abcam), rabbit polyclonal to Col-IV antibody (1:200; Abcam).

### Immunohistochemistry

The renal tissues from vehicle treated mice and HG induced exosomes treated mice were fixed in 4% paraformaldehyde and paraffin-embedded and sectioned for immunohistochemistry (IHC) analysis. Specifically, each section was deparaffinized and dehydrated and subjected to antigen retrieval. The slides were placed in 0.01 mmol/l citrate buffer (pH 6.0) for 15 min in a microwave oven. Endogenous peroxidase activity was blocked with a 3% hydrogen peroxide solution for 10 min at room temperature. After rinsing with PBS, slides were incubated with primary antibodies overnight at 4°C. Sections were then washed in PBS three times, and incubated with secondary antibody for 1 h at 37°C. Finally, the signal was developed with DAB, and all of the slides were counterstained with hematoxylin. Immunohistochemistry was performed with rabbit polyclonal to Fibronectin antibody (1:100; Abcam), rabbit polyclonal to Col-IV antibody (1:100; Abcam).

### Histology

Hematoxylin and eosin (HE) staining was used to examine glomerular mesangial cells proliferation. Mice were treated with vehicle and exosomes at different time points. Then the kidneys were removed and stored in the 4% paraformaldehyde solution. Samples were dehydrated by a gradient of ethanol and embedded in paraffin. Coronal sections were cut at a thickness of 4 μm and standard HE staining was performed. Finally, the sections were observed under light microscopy to evaluate the morphological changes of the kidney.

### Real-time RT-PCR analysis

Total RNA from cells and exosomes was isolated using TRIzol reagent (Sigma) according to the manufacturer's instructions. Relative expression was calculated using the 2−ΔΔCT method and normalized to the expression of β-actin. TGF-β1 Primers: forward: 5′-GCCCTGGATACCAACTATTGCTTCA-3′, reverse: 5′-CAGAAGTTGGCATGGT-3′.

### Enzyme-linked immunosorbent assay

The supernatant was collected for the quantification of main components of ECM (ColIV and FN) using enzyme linked immunosorbent assay (ELISA) according to manufacturer's protocol (CUSABIO, China).

### Statistical analysis

Statistical analysis was performed using SPSS 16.0 software (IBM, USA). Values are expressed as mean±s.d. Differences between groups were calculated using analysis of variance (ANOVA). Differences between two groups were calculated using Tukey's test. *P*<0.05 was defined as significant.
